# “They haven’t asked me. I haven’t told them either”: fertility plan discussions between women living with HIV and healthcare providers in western Ethiopia

**DOI:** 10.1186/s12978-020-00971-2

**Published:** 2020-08-17

**Authors:** Tesfaye Regassa Feyissa, Melissa L. Harris, Deborah Loxton

**Affiliations:** 1 College of Health Science, Wollega University, Nekemte, Oromia Ethiopia; 2 Research Centre for Generational Health and Ageing, School of Medicine and Public Health, Faculty of Health and Medicine, University of Newcastle, University Drive, Callaghan, NSW 2308 Australia

**Keywords:** Ethiopia, HIV, Women, Counselling, Safer conception, Contraception

## Abstract

**Background:**

Despite the importance of women living with HIV (WLHIV) engaging in fertility plan discussions with their healthcare providers (HCPs), little research exists. This study explored perceptions surrounding fertility plan discussions between WLHIV and their HCPs in western Ethiopia, from the perspectives of both women and providers.

**Methods:**

Thirty-one interviews (27 with WLHIV and 4 with HCPs) were conducted at four healthcare facilities in western Ethiopia in 2018. Data were transcribed verbatim and translated into English. Codes and themes were identified using inductive thematic analysis.

**Results:**

There was a discordance between HCPs and WLHIV’s perception regarding the delivery of fertility plan discussions. Only nine of the 27 WLHIV reported they had discussed their personal fertility plans with their HCPs. When discussions did occur, safer conception and contraceptive use were the primary focus. Referrals to mother support groups, adherence counsellors as well as family planning clinics (where they can access reproductive counselling) facilitated fertility discussions. However, lack of initiating discussions by either HCPs or women, high client load and insufficient staffing, and a poor referral system were barriers to discussing fertility plans. Where discussions did occur, barriers to good quality interactions were: (a) lack of recognizing women’s fertility needs; (b) a lack of time and being overworked; (c) mismatched fertility desire among couples; (d) non-disclosure of HIV-positive status to a partner; (e) poor partner involvement; (f) fear of repercussions of disclosing fertility desires to a HCP; and (g) HCPs fear of seroconversion.

**Conclusions:**

Our findings highlight the need for policies and guidelines to support fertility plan discussions. Training of HCPs, provision of non-judgmental and client-centered fertility counselling, improving integration of services along with increased human resources are crucial to counselling provision. Enhancing partner involvement, and supporting and training mother support groups and adherence counsellors in providing fertility plan discussions are crucial to improving safer conception and effective contraceptive use, which helps in having healthy babies and reducing HIV transmission.

## Plain English summary

This study explored perceptions surrounding fertility plan discussions between women living with HIV and healthcare providers in western Ethiopia, from the perspectives of both women and providers. Thirty-one interviews (27 with HIV-positive women and 4 with healthcare providers) were conducted at four healthcare facilities in western Ethiopia in 2018. There was a discordance between healthcare providers and HIV-positive women’s perception regarding the delivery of fertility plan discussions. Only nine of the 27 HIV-positive women reported they had discussed their personal fertility plans with their healthcare providers. When the discussions did occur, safer conception and contraceptive use were the primary focus. Referrals to mother support groups, adherence counsellors as well as family planning clinics facilitated fertility discussions. However, a lack of initiating discussions by either healthcare providers or women, high client load and insufficient staffing, and a poor referral system were barriers to discussing fertility plans. Where discussions did occur, barriers to good quality interactions were: (a) lack of recognizing women’s fertility needs; (b) a lack of time and overworked; (c) mismatched fertility desire among couples; (d) non-disclosure of HIV-positive status to a partner; (e) poor partner involvement; (f) fear of repercussions of disclosing fertility desires to a healthcare provider; and (g) healthcare provider’s fear of seroconversion. Training of healthcare providers, provision of non-judgmental and client-centered fertility counselling along with increased human resources are crucial to counselling provision. Enhancing partner involvement and supporting and training mother support groups and adherence counsellors in providing fertility plan discussions are crucial to improving safer conception and effective contraceptive use.

## Background

Human immunodeficiency virus (HIV) remains one of the main public health problems globally, with 25.7 million people living with the disease in sub-Saharan Africa (SSA) in 2017. Women accounted for 56% of the HIV-positive population in SSA [1, 2]. In this population, antiretroviral therapy (ART) expansion has been associated with prolonged longevity and improved health status [3]. The recent treat-all policy that removes barriers to ART initiation [4] will further reduce morbidity and mortality associated with HIV infections. With improved survival [3, 4] and the reduced rate of mother-to-child transmission of HIV (MTCT) [5], many women living with HIV (WLHIV) have a desire to have children [6]. Given that Ethiopia has among the highest number of people living with HIV (PLHIV) in the world, with approximately 62% of HIV-positive people in Ethiopia being women [7, 8] and a HIV prevalence of 2% among all pregnant women [9], supporting the reproductive care needs of WLHIV is paramount. Importantly, there is an increased need to support preconception care in order to reduce the risk of HIV transmission to both the child [10–12] and partner [13], given the main mode of HIV transmission in Ethiopia is through heterosexual sex (87% of HIV transmissions), followed by MTCT (10% of HIV transmissions) [14].

Preconception care refers to pregnancy planning, reducing unplanned pregnancies, and optimizing maternal and child health prior to pregnancy. Importantly, the reproductive autonomy of WLHIV has received increasing recognition recently [15], despite the history of violating the reproductive rights of WLHIV [16, 17]. Balancing the fertility needs of WLHIV with the prevention of HIV transmission requires multi-dimensional support [15, 18]. This support is required in order to optimize health outcomes for HIV-positive women, their infants and their partners. Effective communication and fertility planning among WLHIV and their healthcare providers (HCPs) is key [19], not only to meet the reproductive goals of HIV-positive people but also to support the prevention of high-risk unintended pregnancies [20, 21]. Effective communication regarding reproductive plans might contribute to a reduced rate of new HIV infections, both among serodiscordant couples and children [10, 19, 22].

Discussing reproductive plans with HCPs can be general, which refers to discussing general issues regarding pregnancy and HIV. Discussing reproductive plans with HCPs can also be personalized, which refers to discussing the client’s own fertility plan [23]. A study from the US and Brazil indicated that general discussions occurred more frequently than personalized fertility plan discussions [23]. To date, particularly in SSA, research regarding fertility plan discussions between HCPs and WLHIV has been limited. Studies have mainly focused on the perspectives of WLHIV in SSA. Two studies from South Africa examined fertility plan discussions among WLHIV [24, 25]. These studies identified that WLHIV seldom received counselling regarding their reproductive plans, although they recognized HCPs as resources for preconception information. Another study from the same country also documented that there were missed opportunities to meet the reproductive healthcare needs of WLHIV due to inadequate reproductive counselling [26]. Inadequate reproductive counselling might be related to the lack of updated and good quality information. Given that little research exists in SSA, particularly in Ethiopia, regarding fertility plan discussions between WLHIV and their HCPs, the need for studies that explore such discussions from both providers and women’s perspectives is high. We therefore conducted interviews with both women and providers to explore discussions between HCPs and WLHIV regarding women’s fertility intentions in western Ethiopia.

## Methods

### Study design and settings

This study employed a phenomenological approach to explore fertility plan discussions between WLHIV and their HCPs in western Ethiopia in August 2018. This framework allowed for the generation of new information about fertility plan discussions between WLHIV and their HCPs [27]. The study was conducted at four healthcare facilities (two hospitals and two health centers) in East and West Wollega Zones. These healthcare facilities provided comprehensive HIV care such as HIV testing and counselling, ART, prevention of mother-to-child transmissions of HIV (PMTCT), and family planning. The healthcare facilities were selected because they had the most clients in the selected zones in western Ethiopia. Further details regarding the study area can be found in Feyissa et al. [29].

### Participant recruitment

Women for this study were identified during the main survey that was conducted prior to the qualitative interviews [29]. On the survey information sheet, women who consented to being surveyed were asked about their willingness to be included in a follow-up qualitative study. Purposive sampling was employed to recruit married women of reproductive age (15–49 years) receiving HIV care at health facilities. Married women were enrolled because the majority of pregnancies and births in Ethiopia occur among married women [28]. These women were eligible for inclusion if they: (i) were willing to participate in the qualitative interviews [29]; (ii) could provide information regarding their reproductive plans during the interview; and (iii) could provide information regarding their reproductive plan discussions with HCPs during the interview. Women who were pregnant, breastfeeding and attempting pregnancy as well as using or not contraception were eligible to participate. However, WLHIV who were critically ill were excluded due to the difficulty of recruiting them at inpatient units and potential difficulty in obtaining responses. No pre-existing relationship existed between the interviewers and participants. Maximum variation sampling was considered for in-depth interviews to maximize the diversity of information relevant to the research question. Furthermore, HCPs were included to understand provider’s perspectives regarding fertility plan discussions with WLHIV. HCPs were nurses, physicians, midwives or health officers (clinician) working [30] and performing within the scope of their practice in HIV clinics of the selected health facilities [31, 32]. An initial sample size of ten WLHIV and two HCPs was set. However, the final sample size was determined by saturation of information, that is when recurrent patterns become evident in the participants’ narrations [33].

### Ethical considerations

Ethical approval was granted by the Human Research Ethics Committee (HREC) of The University of Newcastle, Australia (H-2017-0289), and the Oromia Regional State Health Bureau Research Ethics Committee, Ethiopia (BEFO/HBISH/1–16/257). All participants provided informed consent for the interview and for their interviews to be audio recorded. Further information regarding the ethical considerations for this project can be found in Feyissa et al. [29].

### Data collection

Because of the sensitive nature of the information, two female nurses with master’s degrees were trained by the first author (TF) to conduct the interviews with the WLHIV. This was done to help participants feel more comfortable in discussing their fertility needs. Thus, female interviewers who had previous experience in HIV training conducted the face-to-face interviews with WLHIV after 5 days of training and pilot testing to reduce the possible impact of gender. Interviews with HCPs were conducted by the first author (TF), a PhD candidate with a background in nursing and postgraduate training in public health. None of the interviewers had ever worked at the selected health facilities. The motivation for this research arose from the desire to understand women’s fertility plan discussions with a focus on personalized fertility plan discussions. The researchers used open-ended questions to enrich the depth of information. The interviewers followed the direction of the participants to get a deep understanding of their fertility discussions. No one else was present during the interviews besides the participant and the researcher. All interviews were conducted in a private room in the HIV clinic in the local language, Oromo, using semi-structured guides. The guide for the interviews with the HCPs addressed perceptions regarding providing fertility plan discussions with WLHIV, what they discuss about women’s fertility plans, and what stops them from having or initiating such discussions. The interview guide for WLHIV contained questions regarding plans to have a child, discussions about fertility plans, pregnancy planning experiences and prevention of HIV to a child and/or a partner. All interviews were audio recorded. The interviews with HCPs took between 33 and 50 min (median 41 min), while the interviews with WLHIV took between 15 and 57 min (median 29 min).

### Data processing and analysis

Interviews were transcribed verbatim in the local language and then translated into English by two healthcare professionals who have master’s degrees. All transcripts were downloaded into the qualitative management program NVIVO (Version 12. QSR International; 2019) for analysis. Thematic analysis was used as it is not wedded to a particular epistemology. Braun and Clarke’s (2006) thematic analysis principles [34] were applied as a guide to elucidate the themes. This method provided a guide for examining the perspectives of participants, highlighting similarities and differences and generating unanticipated insights. It was also considered suitable due to the exploratory nature of the inquiry (see Supplementary File 1 for further details). Selected quotes that best described the various categories were included to illustrate major findings while avoiding redundancy. During quoting, WLHIV were identified by code (W#1-W#27), age, number of children they had, number of children desired in the future and whether they discussed their *personal fertility plans* with their HCPs (discussed/not discussed). HCPs were identified by code (HCP#1-HCP#4) to protect their privacy (Table 2 and Table 3).

## Results

### Participant characteristics and summary of themes

Thirty-one interviews (27 with WLHIV and 4 with HCPs) were conducted in western Ethiopia. One HCP from each healthcare facility providing routine HIV-care at each HIV clinic (four in total) was interviewed. No women or HCPs declined to take part in the interviews. All WLHIV were using ART at the time of the interview. Participating WLHIV included five women who were pregnant, six who were breastfeeding and four who were attempting pregnancy as well as seven who were using contraception and three who were not using contraception. Nine women had three or more children. Five women desired one child in the future, while seven women desired two or more children in the future. The age of the WLHIV ranged from 18 to 38 years, with a mean of 28 years (see Table 1). Two nurses and two health officers (clinicians) who provided direct care for people living with HIV were included. The HCPs aged between 33 to 48 years and had between 15 and 30 years of work experience as healthcare professionals.

**Table 1:** Socio-demographic characteristics of women living with HIV included in the interviews in western Ethiopia, 2018

Characteristics	Category	*N* = 27
Health facility	Hospitals	13
	Health centers	14
Age (in years)	Mean age	28
	Range	18–38
Schooling	No formal education	3
	Primary education	16
	Secondary and above education	8
Number of children	None	3
	1–2	15
	3 or more	9
Future fertility intention	None	15
	1	5
	2 or more	7
Employment status	Small business owner/ trader	9
	Housewife/no job	10
	Government/ Non-government employee	3
	Farmer	5
Monthly family income	Less than 1500 Ethiopian Birr	15
	≥1500 Ethiopian Birr	12
Time since HIV diagnosis	≤ 5 years	9
	5 to < 10 years	7
	≥10 Years	11
Time on ART	≤ 5 years	10
	5 to < 10 years	9
	≥10 Years	8
Status of women	Pregnant	5
	Breastfeeding	6
	Using contraception	7
	Recent abortion	1
	Not using contraception	3
	Immunization	1
	Attempting pregnancy	4

Four major themes were identified: (a) *Discussing fertility plans* described perceptions around discussions between WLHIV and their HCPs regarding fertility plans; (b) *Facilitators to discussing fertility plans* presented contexts that enabled fertility plan discussions to occur; (c) *Barriers to discussions* highlighted factors that prevented fertility plan discussions from occurring; and (d) *Barriers to good quality discussions* identified the factors that hindered the quality of discussions.

### Discussing fertility plans

This theme contextualized the providers and women’s perceptions of discussing WLHIV’s fertility plans. Reflecting on fertility plan discussion, this theme also described the primary focus of fertility plan discussions.

A discordance in perception between the women and providers in the delivery of fertility plan discussions was noted. Only 9 of the 27 WLHIV reported having discussed their future personal fertility plans with their HCPs however all HCPs said the discussions occurred with WLHIV during almost all visits. Importantly, for the women who did engage in discussion with their providers, both women who wanted and did not want children had discussed their personal fertility plans with their HCPs. A woman who wanted to have children said: “*They [HCP] counselled me that I can have a child” (W#5, 26 years, had one child, three more children desired, discussed).* HCPs reported that there were more frequent fertility discussions compared to the women (Table 2, quote 1)*.* The reason for more frequent personal fertility plan discussions according to HCPs was that the ART follow-up chart required the provider to ask about the fertility desires of WLHIV (Table 2, quote 2)*.*

**Table 2:** Healthcare providers’ perception regarding fertility plan discussions with women living with HIV in western Ethiopia

Themes/categories	Quote Code	Quote
Discussing fertility plans		
	1	They [women] discuss with us when they visit our center (HCP#2).
	2	Moreover, in the treatment rooms, patients are asked information on their desires on the ART follow-up chart. This is important to know the status of our patients (HCP#4).
Safer conception strategies	3	To have a child, viral load should be less than 1000 and CD4 [count] should be greater than 350 [cells/μL] (HCP#1).
	4	If their weight is low, in turn, there is the possibility that their CD4 is low, we advise them not to have a child. We provide them counselling if their CD4 [count] is less than 500 and the viral load above 1000. Besides, we encourage both [a wife and husband] not to have a child during initial HIV drug use and low CD4 count (HCP#4).
	5	The main thing is they should not have OI [opportunistic infections] and women’s clinical stage is assessed by providers especially for those [with] clinical stages of 3 and 4 (HCP#3).
	6	Anyhow, they have to wait for the ovulation period in order to have sexual intercourse without condoms, in turn, to get a child. Afterwards, they have to use condoms regularly. In principle, a healthcare worker cannot counsel sex without condoms with a HIV-infected one. It is a major way of its spread. Accordingly, health worker’s advice is telling the truth behind sexual [condomless] engagement. The choice and the move for [decision to have] a child through having [unprotected] sex with a HIV-positive person depends on the decision of the partner, or one of them (HCP#3).
	7	Since she was on ART for a long period and her CD4 [count] was also not in clinical-stage condition, her child was not reactive (HCP#3).
Contraceptive discussions	8	We ask their choice [s], inform them the available types of contraception and ask [them] any complaint regarding previously used contraception. If it caused bleeding, prolonged [menstrual] period, we change for them by asking the suitable contraception. [But] we can’t give them oral contraceptives … We counsel them not to use hormonal contraception [OCP], since this type of contraceptive methods may weaken ART they use (HCP#1).
	9	For long-acting family planning service [s], we do not restrict women and women can choose freely (HCP#4).
	10	Among contraception methods, they can use condoms and IUD [s] since they lack hormone [s]. ART drugs have an impact on hormonal contraception [OCP] and can cause pregnancy. Injectables [short-acting] and implants are also possible [to use] (HCP#1).
	11	For people living with HIV, contraceptive methods like pills or depo or implants only are not recommended. Because there are opportunistic infections; viral load can be transmitted; HIV of the husband can be transmitted [cross infection] (HCP#3).
	12	We provide short-acting contraception at our clinic [HIV clinic], whereas long term contraception is provided at family planning unit [s] (HCP#4).
	13	Some clients say, ‘I become pregnant while I’m using depo (injectables)’, while others say, ‘I was using implant during my conception’. We counsel them there may be a failure of contraception (HCP#3).

There was incongruence between HCPs and WLHIV regarding the delivery of personalized fertility plan discussions. Some barriers prevented discussing fertility plans. In addition, several factors hindered good quality discussions between HCPs and WLHIV. When fertility plan discussions occurred, they often broached the subjects of safer conception strategies and contraceptive use.

#### Safer conception strategies

This sub-theme focused on strategies that were discussed between WLHIV and their HCPs to minimize risks when attempting pregnancy. Although there were inconsistencies regarding these risk-minimizing strategies, higher CD4 count, lower viral load, not at AIDS stage 3 or 4, timed unprotected sex and using ART for a longer period were the safe conception strategies that were discussed between WLHIV and their HCPs. Discussions of such strategies were mainly reported by HCPs.

HCPs indicated that CD4 counts above 350 and viral loads below 1000 cells/μL were important preconception preparations (Table 2, quote 3). Another HCP reported different cut-off points for CD4 count (500 cells/μL) to attempt pregnancy (Table 2, quote 4). This view was supported by the women. A woman was told by a provider not to attempt pregnancy because her CD4 count was 400 or 500: “*They told me ‘if your CD4 [count] is 400 or 500, you can’t have a child’. My CD4 [count] was 600 or 700, I had a child” (W#14, 24 years, had two children, two more desired, discussed).* Importantly, being free of opportunistic infections particularly not being at AIDS stage 3 or 4 were also mentioned as criteria to attempt pregnancy by a HCP (Table 2, quote 5)*.*

A few WLHIV and their HCPs reported that timed unprotected sex was a strategy to achieve pregnancy safely. Inconsistencies regarding the timings of this strategy however were reported. A woman reported that it was having condomless sexual intercourse for 5 days after cessation of menstrual period: *“The healthcare provider advised me to have periodic sexual intercourse for five days after cessation of menstrual blood flow. To use condoms other time” (W#11, 30 years, had no children, four children desired, discussed).* Meanwhile, another participant stated that timed condomless sex was after 10 days of menstrual bleeding through checking both viral load and CD4 count: “*By checking CD4 [count] and what is called viral load, then, we [women] can have sex without condoms after 10 days of menstrual bleeding until fertilization of prepared eggs” (W#19, 38 years, had four children, no more desired, discussed).* Although timed unprotected sex was one strategy for safer conception as noted by the women, HCPs reported that they do not advise this strategy. HCPs emphasized HIV transmission risks associated with timed condomless sex. However, HCPs reported that the final decision to attempt pregnancy through timed condomless sex was in the hands of HIV-positive people (Table 2, quote 6).

Some WLHIV reported their recent pregnancy was intended and they stopped using condoms to have a child. One participant explained: “*Most of the time, we were using contraception. We stopped using condoms when we decided to have children” (W#4, 20 years, had one child, two more desired, not discussed).* This was confirmed by another woman who said: “*I was taking Depo Provera. I discontinued [Depo] to have a child” (W#8, 32 years, had three children, no more desired, discussed)*.

Finally, the WLHIV wanted children and it was a priority that their newborn children are HIV free. This contextualized the demand for safer conception. A woman who did not discuss her personal plans said: “*Both of my children are checked and they are free” (W#16, 22 years, had two children, no more desired, not discussed).* Furthermore, a HCP explained that using ART for a longer period and high CD4 was associated with having a HIV-free child (Table 2, quote 7). WLHIV who had prior experience of having a HIV-negative child had reduced their worry and favoured trying conception.

*I was very much worried about my first child. I was thinking how a person living with HIV can have a baby [HIV-free] … But after I had my first child, then I started to think that it is possible, my knowledge about HIV also increased. I am thinking that it is possible to have a HIV-free child (W#15, 30 years, had two children, one more desired, not discussed).*

Safer conception strategies for WLHIV who intended to have children focused on optimal CD4 count, viral load and timed unprotected sex. WLHIV who did not intend to have children need contraceptive discussions.

#### Contraceptive discussions

This sub-theme focused on contraceptive discussions, the roles HCPs played in contraceptive choice, availability of contraceptive methods and demand for contraceptive discussions. Both WLHIV and HCPs reported multiple contraceptive methods were available. Long-acting reversible contraception (LARC) and condoms were preferred methods and there seemed to be a lack of contraceptive decision-making autonomy for the women, with contraceptive choice dictated by the HCPs. According to WLHIV, contraceptive methods such as injectables were not recommended because it was thought that it ‘softens their bones’. A woman said: *“They advise us on contraception. There is [are] contraception of 5 years, 3 years, 12 years, pills and injection types. Because it [injectable] can soften bone, the injection type of contraceptive method is not advised. We [women] are using the 3 years and 5 years types mostly. They [HCPs] also select which is suitable for us” (W#19, 38 years, had four children, no more desired, discussed).*

In contrast, the HCPs reported that there was shared decision-making regarding contraception and that they informed women of all available contraceptive types except for some that might interfere with HIV-drugs. In particular, HCPs did not recommend the use of oral contraceptives due to the possibility of drug-drug interaction with HIV-drugs and if adverse side effects were identified with their previous use (Table 2, quote 8).

According to the HCPs, it seemed that there was no restriction among long-acting contraception (Table 2, quote 9)*.* However, another HCP reported a preference for hormone lacking contraception, such as Intrauterine Devices (IUD) and condoms (Table 2, quote 10)*.* Dual methods were promoted by HCPs to prevent opportunistic infections and HIV transmissions (Table 2, quote 11).

*Because I am living with HIV, I discussed what type of contraception was favourable for me. I am using contraception which is inserted to the arm … In addition, I am using condoms (W#11, 30 years, had no children, four children desired, discussed).*

A lack of access to effective contraception was noted by HCPs with LARC only available at family planning clinics (not available at HIV-clinics) although these methods were recommended by HCPs (Table 2, quote 12). Despite this, WLHIV reported using both short-acting and long-acting contraception. A woman stated: “*I am using which [contraception] is placed in the womb [IUD]” (W#2, 18 years, had one child, no more desired, not discussed)* while another participant reported being hypertensive and was advised by a HCP to shift to tubal ligation.

*They advised me. It is risky to have additional children with this blood pressure [hypertension] and I decided to have ligation [tubal] … I did so [tubal ligation] (W#22, 29 years, had five children, no more desired, discussed).*

The importance of personalized client-provider discussions regarding the choice of contraception was reported by the women: “*We discussed, but I said it is enough for me … I am using contraception that is for five years [implant]” (W#10, 28 years, had two children, no more desired, discussed).* Some other participants expressed frustration regarding their current method of contraception and need for more effective methods. In particular, a woman expressed: “*I am using the injectable which is for three months. Condoms may break” (W#17, 25 years, had two children, no more desired, not discussed).* However, there were women who were confident in using methods such as condoms: “*There is no nagging; we use condoms” (W#13, 22 years, had one child, three more desired, not discussed).* The partner being unsupportive of using condoms was reported as a reason not to use condoms: “*We rarely use condoms because he does not say ok (W#15, 30 years, had two children, one more desired, not discussed).* Gaps regarding availability and concerns with current contraceptive methods might have resulted in unintended pregnancies.

Despite women reporting the use of contraception, recent unintended pregnancies were reported by the women being interviewed. These were often described as being a result of unmet needs for contraception, contraceptive failure or contraceptive misuse.

*Yes, it is without plan. I didn't want to get pregnant. I, myself, is like this [HIV-positive] … it was difficult to terminate after the pregnancy was big. Hence, I had this child (W#9, 25 years, had two children, no more desired, discussed).*

*I know but I took and was taught to use condoms. However, one day I had sexual intercourse without a condom, then conceived (W#10, 28 years, two children, no desire, discussed).*

HCPs reported their frustration with the unintended pregnancies of their clients and counselled on the effectiveness of contraception (Table 2, quote 13)*.*WLHIV held inaccurate knowledge of how to identify days with peak fertility to practice timed unprotected sex as well as CD4 level and viral load. There has been inconsistencies and frustration regarding contraception by both WLHIV and HCPs.

### Facilitators to discussing reproductive plans

This theme identified contexts perceived by HCPs that facilitated fertility plan discussions, but these facilitators were not identified by WLHIV. These factors encompassed the availability of mother support groups, adherence supporters, referral to other units, family planning clinics, and morning health education sessions.

Referrals to other units and peer educators (mother support groups^1^ and adherence supporters^2^) were identified by HCPs as important facilitators for discussing personal reproductive plans. HCPs explained there were referrals to mother support groups along with plans (Table 3, quote 14). Referrals to the two mum-to-mum supporters were also to avoid a long waiting time that could be created on other patients at HIV clinic due to high client load. The roles of mother support groups included social support, advice regarding pregnancy care, postnatal care, newborn care, laboratory investigations, as well as education on other related health affairs (Table 3, quote 15 and quote 16).

**Table 3:** Healthcare providers’ perception regarding facilitators and barriers to fertility plan discussions with women living with HIV in western Ethiopia

Themes	Quote Code	Quote
Facilitators to discussing reproductive plans		
	14	We prepare a plan and send to the mum-to-mum group [s]. Mum-to-mum group [s] give them a support when they [women] need to get pregnant, follow them and find them even if they missed during follow-up (HCP#1).
	15	The mother support carers are working with an NGO [non-governmental organization named ICAP-The International Center for AIDS Care and Treatment Programs]. At this center, they are providing close counselling for pregnant women. In addition to medical consultation, they prepare coffee and tea ceremony. The mother support carers provide advice for pregnant women related to care during pregnancy, post-delivery care for children, medical examination, DBS [Dry Blood Sample] for children, and virological test [s]. Hence, the two women [mum-to-mum] are shouldering those duties … Furthermore, they are arranging education service program [s] (HCP#2).
	16	We spend 30 min with a single patient, then adherence supporters counsel them [HIV-positive women] (HCP#3).
	17	We refer them for family planning service [to family planning clinics], particularly for long-acting [contraception] … By the way, we are providing health education to enhance awareness during every morning on different topics (HCP#4).
Barriers to discussions		
High client load and insufficient staffing	18	Human power has also its own impact as ART guideline recommends 20 patients per day, but we have about 45, 40, or 35 [per day]...The shortage of human resource [s] is creating a challenge to the implementation of policy. The room at which we are providing a service for HIV-positive people often gets busy (HCP#3).
A poor referral system	19	Moreover, we refer them to family planning clinic... During referral time, there are some dropouts (HCP#4).
Barriers to good quality discussions		
Lack of recognizing women’s fertility needs	20	Not many, they are about 5–10 person out of 100 (HCP#1).
	21	If they insist on having a child, we discuss with them on the possible opportunities [strategies] (HCP#4).
A lack of time and being overworked	22	There is high client load … So, this makes [it] difficult to provide a complete service because it takes time. A lot of patients are waiting for me [waiting time] while I’m discussing with a single client for some time...It is hardly possible for one worker to serve [all] customers. There are also additional duties as workers provide services in an integrated approach. [There is] duty of registration, a worker is doing throughout a day like a report writer (HCP#3).
Mismatched fertility desires among couples	23	He [man] wants to see his offspring before death and force woman to have a child. Less frequently, a woman also wants to have a child because she does not have a child before (HCP#2).
	24	They [couple] are opposing each other. She didn’t need to have a child, because he drank alcohol, and didn’t believe her, he is [HIV] positive and she is [HIV] negative (HCP#1).
Nondisclosure of HIV-status to a partner	25	Another challenge is about disclosure. It is difficult to disclose the status of the patients, particularly beyond the existing ethics and regulation and their interests. In some cases, a husband knows; in other case [s], a woman knows ... Some women do not want their personal health status to be revealed to their husbands being fear of the possible consequences such as separation in which they may face difficulty in [future] living condition and their lives … She might be pregnant during this time (HCP#4).
Poor partner involvement	26	Women [some] who are tested positive through our clinic do not tell their husband. But there are cases where we invite the husband and he gets tested. In some cases, he refuses to come. On the other side, there is a situation in which husbands bring their wives to the clinic for a test [HIV] … Therefore, their husbands have to receive advice at our center to use condoms properly. Those [women] who have husbands want their consensus for contraceptive use (HCP#2).
	27	For instance, if a woman is HIV-negative and a man is HIV-positive, you advise the use of condoms. In practice, there is a situation he has sex without condoms. He says, ‘let me live then let die’. Thus, he has sex without condoms. On the other hand, if a woman is HIV positive and a husband is HIV negative, there is a condition in which the husband leaves her or gets separated (HCP#2).
	28	In fact, most of the time, they were required to receive close advice at the center. But, a husband sometimes says, ‘I am free and but it is up to you’ (for discussion) (HCP#2).
	29	There are people who have two or three children while being discordant... We tell the HIV-negative person, there is the possibility to acquire HIV when having sex without condoms (HCP#3).
	30	In the process, we observe the discordant converting to positive. Some of them [wrongly] believe that they could not be infected because of their blood type (HCP#3).
	31	Sometimes they [couple] come together, other time [s] the woman alone (HCP#1).
Fear of repercussions of disclosing fertility desires to a HCP	32	There are people who desire to have a child, but fear to disclose (HCP#1).
	33	We have been advising her not to get pregnant. She may fear to inform us since she gets pregnant against our counselling … First, if a child gets HIV-infected, it is a burden to a family. Secondly, a child born with HIV suffers a lot throughout his life including taking drugs. So, we advise couples [family] not to have a child (HCP#2).
	34	Most of the time, those women who lack or have one child only come with their partner and discuss with us. Those having 3 or more children do not disclose [intention to have a child] for the matter they may lack permission (HCP#3).
	35	She said ‘I feared them [HCPs] since I told them I have neither a husband nor [boy] a friend’. Secondly, the reason for this case was the short contact time with the provider (HCP#3).
	36	Seldom, there is a possibility of getting pregnant while we are counselling them not to have a child. When we ask them ‘why they do like that; why you don’t tell us?’ Their response is that ‘you advised us to delay pregnancies and you may not accept but we wanted to have a child’. The decision behind at large was due to their fear of pressure from providers (HCP#4).
HCPs fear of seroconversion	37	In fact, we strongly promote practice of safe sex with condoms to all our patients, in particular for discordant in our institution. You [HCP] need to avoid any complaint against us if they get seroconverted (HCP#3).

Furthermore, HCPs reported that they referred WLHIV to family planning clinics for LARC methods. Morning health education sessions also helped to create awareness of different topics (Table 3, quote 17).

### Barriers to discussions

This theme highlighted contexts perceived by HCPs and WLHIV that prevented fertility plan discussions. The majority (18 of the 27) of WLHIV reported that they hadn’t discussed their personal fertility plans with their HCPs. According to the perspectives of both WLHIV and HCPs, lack of initiating discussions, high client load and insufficient staffing, and a poor referral system were barriers to fertility discussions.

#### Lack of initiating discussions by either HCPs or WLHIV

This sub-theme identified that the majority of women reported there was no discussion regarding their personal fertility plans due to the lack of initiating conversations by either HCPs or the women themselves on the issues of childbearing. There were uncertainties about who should initiate the conversations. One woman’s statement summarized this: *“They haven’t asked me. I haven’t told them either” (W#1, 36 years, had two children, two more desired, not discussed).* Women also reported that there was a delay in having discussions with their providers (though attending ART for more than a year), but the HCPs said they were going to talk to them: “*… we are going to talk to a doctor on this issue since I don’t have knowledge about this” (W#13, 22 years, had one child, three more desired, not discussed).* Although the timeline to discuss their fertility plans was not clear, a woman reported readiness to accept the recommendations of HCPs: *“If they say you can have, we will have an additional child” (W#26, 33 years, had two children, one more desired, not discussed).*

#### High client load and insufficient staffing

This sub-theme contextualized high client load and insufficient staffing as a barrier to discussing fertility plans. Despite the ability to provide referrals to other units, high client load, mainly explained in relation to insufficient staffing, was noted as a critical barrier to discussing personal fertility plans. This resulted in an inability to translate policy into action (Table 3, quote 18)*.* This was also supported by a woman’s report that there was a lack of access to have discussions because their provider was occupied most of the time: *“The healthcare workers are occupied most of the time” (W#27, 34 years, had four children, no more desired, not discussed)*.

#### A poor referral system

A poor referral system involved missed opportunities when women were referred to family planning clinics. Although referrals to other units such as family planning clinics were identified as facilitators to fertility plan discussions, HCPs reported there were missed opportunities during referral because some women went home rather than to the family planning clinics (Table 3, quote 19). The HCPs reported that they referred women to other units but some women could not get into having discussions at the referral units i.e. ‘falling through the cracks’.

### Barriers to good quality discussions

This theme highlighted contexts perceived by HCPs and WLHIV that prevented the conducting of good quality fertility discussions. In this context, a good quality discussion refers to actively and fully discussing and disclosing their personalized concerns and preferences to their HCPs. This further involves enhanced communication by HCPs to assess women’s concerns related to fertility accurately and having personalized discussions that were relevant to WLHIV.

#### Lack of recognizing women’s fertility needs

Exploring this sub-theme in the context of the lack of recognizing women’s fertility needs highlighted the discordance in the rate of fertility desire reported by providers and WLHIV. Among WLHIV, nearly half (12 of the 27) of women expressed a desire to have a child in the future and the potential to transfer the disease was at the forefront of their concerns. The lack of recognizing fertility needs by HCPs was contextualized in that HCPs reported that only a few women (5–10 women out of a hundred women) desired a child (Table 3, quote 20). This lack of recognition of fertility needs might lead to women not receiving the counselling needed according to their fertility plans. This was explained by some WLHIV as having a child against permission (advice) of their providers: “*It is not permitted [to have a child]. It has risk but I wanted to have” (W#18, 35 years, had four children, no more desired, not discussed).* The HCPs also stated that discussions about having children were made if the WLHIV insisted on having children (Table 3, quote 21).

The differences in the rate of fertility desire reported by HCPs and WLHIV might have resulted in poor (inaccurate) information based on client’s own fertility needs and caused women fear to disclose their fertility needs.

#### A lack of time and being overworked

This sub-theme contextualized not allowing enough time for discussions because providers were rushing during discussions on different topics and focused on general fertility discussions as opposed to personalized. To reduce waiting for the next patient, booking times were too short, discussions were shallow and did not allow for consults beyond HIV management. According to the provider’s perspectives, the reasons for these discussions that were shallow was related to a shortage of human power and high client load. Service integrations (HIV services and other reproductive health services for a client at the HIV-clinics) also demanded more time (time-consuming and creates longer waiting time). Parallel activities of the clinic, like recording and preparing reports added more working time on HCPs so that they rushed (Table 3, quote 22).

#### Mismatched fertility desires among couples

This sub-theme focused on couples having discordant fertility needs (only one of them desiring to have a child), which hindered full disclosure of concerns, and prevented them from discussing their fertility plans fully. In the context of mismatched fertility desires, a woman expressed that her partner was not concerned about fertility issues and was just pushing her to have more children: “*Yes, I have told him. He simply said it is good. It is not his concern whatever number [of children] I have” (W#3, 30 years, had two children, no more desired, not discussed).* In the context of HIV-positive people, a HCP explained that there was a pressure mainly from men that women bear more children (Table 3, quote 23)*.* Additional concern by HCPs was that when the mismatched fertility desire was among serodiscordant couples (Table 3, quote 24)*.*

#### Nondisclosure of HIV-status to a partner

Non-disclosure of HIV-positive status to a partner was another barrier to good quality interactions. Disclosing HIV status to a partner took years (5 years) in some cases. Discussing fertility plans with a couple in relation to HIV was difficult during a longer nondisclosure period. A woman said: *“I did not tell this to my husband. It is just one year since he tested but I have known my status for the past six years. I started using medications six years ago but it is just one year for him” (W#16, 22 years, had two children, no more desired, not discussed).* Due to ethical reasons, HCPs found it challenging to disclose their client’s HIV-status to their client’s partner when their clients did not want their partner to know about it (Table 3, quote 25).

#### Poor partner involvement

Poor partner involvement was a barrier to good quality fertility discussions. Contraceptive counselling is less effective when the partner is not involved. Reasons for low partner involvement were unwillingness to provide consent, refusal to visit the health facility and failure to accept the advice of HCPs. This was reported by women: “*The providers advise me but he [partner] wants without protection. We use as such [without condoms though HIV-negative]” (W#27, 34 years, had four children, no more desired, not discussed).* HCPs also expressed that some partners were not coming to health facilities when invited for counselling (Table 3, quote 26).

Furthermore, being serodiscordant affected fertility discussions by affecting marital relationships, but it mainly depended on who was HIV-positive. HCPs reported that if men were HIV-positive and women were HIV-negative, the couples stayed married. If the situation was reversed, the male partner filed for divorce. If the men were HIV-positive, there was a greater chance that they had sex without condoms if they maintained their marital relationship (Table 3, quote 27).

The HCPs explained that some HIV-negative men who maintained relationships believed that discussing fertility plan was women’s business (Table 3, quote 28)*.* Furthermore, there were some myths about HIV infections, such as the belief that one can be unsusceptible to HIV due to blood type. A woman said: “*They say their blood type is different and that is why they don’t get caught” (W#15, 30 years, had two children, one more desired, not discussed).* A HCP echoed the perception regarding the myth about HIV infections (Table 3, quote 29)*.*

Prevention of HIV transmission to a partner was practised by using drugs and condoms. However, seroconversions were reported by both WLHIV and HCPs. A WLHIV who was HIV-positive at marriage while her husband was HIV-negative but acquired it later explained the situation.

*He was free when we got married. I told him that I was HIV-positive. He said I want to live with you whatever happened. He was free after we had the first daughter. He acquired the disease [HIV] after that and is now receiving his drug from health center … If he used condoms, it was possible to prevent transmission [HIV]. I took [condoms] home but he refused to use condoms (W#3, 30 years, had two children, no more desired, not discussed).*

Misinformation among discordant couples in which the HIV-negative person said they could not acquire HIV due to their blood type might lead to HIV infections (Table 3, quote 30)*.* At the other end of the spectrum, there were couples who came for discussions. The HCPs tried to communicate and deal with the male partner (Table 3, quote 31). Some partners accept the advice of their HCPs. A woman said: “*He was called by the healthcare provider and told that we can’t have an additional child. Therefore, he doesn’t comment and says to me whether we should have a child or not (W#22, 29 years, had five children, no more desired, discussed).*

#### Fear of repercussions of disclosing fertility desires to a HCP

This sub-theme encompassed fear of repercussions due to disclosing their fertility desire purposely because fertility counselling by HCPs is mainly directed towards not having children. Fear of repercussions did not stop at the consequences of disclosing fertility desire before a pregnancy, but also extended to fear of the consequences of disclosing a pregnancy during its early stages. The HCPs wanted WLHIV to raise their existing children. In fear of HCP’s judgement, WLHIV preferred not to disclose their desires. A woman who discussed her fertility plan but was advised to care for the children she had said: *“They advise me to care for these children ... Health providers do not allow having children” (W#18, 35 years, had four children, no more desired, discussed).* This was echoed by another woman: “*Health care providers advised me not to have an additional child” (W#25, 28 years, had two children, one more desired, not discussed).*

Directed counselling from providers caused women to fear disclosing their fertility desires (Table 3, quote 32)*.* The concern of HCPs was mainly HIV-related, such as risk of transmission of HIV to the child (Table 3, quote 33)*.* Another HCP reported that counselling to avoid having children depends on the circumstances of women, i.e., the number of children they had; those women who had 2 or 3 children were not disclosing their fertility desires because of a possible lack of permission to have a child (Table 3, quote 34).

Nondisclosure of pregnancy during the early stages was related to feelings of embarrassment in reporting to providers. HCPs gave examples of experiences they had in HIV-clinics. A HCP said a woman who reported she had no sexual partner previously feared to disclose her pregnancy (Table 3, quote 35)*.* HCPs also reported that WLHIV who were advised to delay pregnancies did not disclose when they fell pregnant because of a fear of it not being accepted (Table 3, quote 36).

#### HCPs fear of seroconversion

This sub-theme was about providers imposing their concerns on women rather than providing client-centered fertility counselling. Fear dominated the lack of engagement in reproductive discussion because of scientific facts behind seroconversion. Counselling by HCPs to serodiscordant couples was mainly focused on advice to avoid having children because of fear of seroconversions. HCPs perceived that if the HIV-negative partner seroconverted while trying to conceive, complaints (blaming the HCPs that it was related to their advice and they could have done something better) might be directed towards them (Table 3, quote 37)*.*

## Discussion

This study provided qualitative insights into fertility plan discussions between WLHIV and their HCPs in western Ethiopia from both a HCP and patient perspective. When fertility plan discussions did occur, safer conception strategies and contraceptive use were the primary focus. Creating understanding among HCPs and WLHIV regarding optimal CD4 count, viral load, timed unprotected sex, and effective contraception was important. There were incongruences between client and provider perceptions regarding fertility plan discussions delivery and there were uncertainties about who should initiate the conversations. Barriers to good quality interactions might have caused information to either not be provided in a way that is understood by clients, and/or that is not retained by clients. Fear dominated the lack of engagement in reproductive plan discussions. WLHIV had fears about repercussions of disclosing their fertility desire to a HCP while providers feared seroconversion among serodiscordant couples. Supporting the disclosure of HIV-status to partners could also enhance the effectiveness of fertility plan discussions. Directed counselling towards avoiding having children might also be the reason for lack of knowledge regarding safer conception strategies.

Although the importance of client-provider discussions in meeting the reproductive needs of WLHIV has been documented [19, 35], there was a difference between HCPs and WLHIV’s perception of the delivery of fertility plan discussions. While HIV-positive women reported fertility plan discussions were rare, HCPs reported frequent fertility plan discussions in our study. The barriers to good quality discussions might have resulted in inadequate understanding among WLHIV about safer conception and contraceptive methods [36, 37]. If WLHIV receive quality reproductive information, they might make better-informed decisions and feel more in control of their fertility plan decisions [29]. Improving both number and quality of trained human resources to avoid rushing and effective service integration is therefore very helpful in improving the quality of fertility plan discussions [38]. Involving women in their preconception planning through discussions and non-judgemental care is also very important.

Although safer conception strategies are required to optimize health outcomes of WLHIV, their infants and their partners [39], our findings highlighted that safer conception discussions were mainly reported by HCPs. This included clinical readiness, such as a higher CD4 count, a lower viral load, and not being at AIDS stage 3 or 4. There were inconsistencies reported regarding the ideal CD4 level at which WLHIV should attempt to get pregnant. Previous studies have shown that a higher CD4 count and a lower viral load benefits both women and children [40, 41]. It is therefore vital to create a shared understanding among both HCPs and WLHIV regarding the optimal CD4 count and viral load level prior to attempting pregnancies. Shared decision-making in fertility plan counselling might be valuable in order to improve patient satisfaction [42]. Timed unprotected sex, which was also identified in the current study, is another safer conception strategy [43, 44] but availing of other assisted reproductive choices are also relevant [45]. Importantly, full package safer conception discussions including ART adherence, pre-exposure prophylaxis for HIV-negative partners, regular fertility and sexual behaviour tracking, and counselling on self-insemination [39] are important, given WLHIV were open to having children in our study.

In the recent consensus statement, there were gaps regarding safer conception implementation [10]. There was no policy supporting safer conception strategies, especially in Ethiopia [45]. For example, the PMTCT guideline in Ethiopia mainly focuses on HIV-drugs among pregnant women [46]. Although HIV-drugs among pregnant women are crucial, prevention of unplanned pregnancies [47] and preconception planning [10, 48] can be more effective in preventing HIV among children. Taken together, guidelines and policies for safer conceptions, shared preconception planning, as well as provider-training can maximize the benefits of preconception care.

Our study identified that contraceptive use was a contentious point of discussion with differing perspectives among HCPs and patients. HCPs had some preference for certain contraceptive methods during counselling (e.g., IUDs and condoms). Importantly, referral to family planning clinics facilitated fertility discussions. However, our study found there were missed opportunities during the referral system as HCPs reported that a number of women failed to take up the referrals to other organisations such as Family Planning. Improving the quality of the referral system through an appropriate regular tracking system for the missed opportunities and dropouts and acquisition of feedback of referrals might have a better yield for contraception discussion and use. Furthermore, the recent unintended pregnancies were related to non-use, misuse of contraceptives [21], and partners being uncooperative in using contraception (e.g., condoms). Therefore, strengthening family planning programs and male involvement in family planning have significance in improving contraceptive use.

Importantly, we identified that referrals to peer educators (both mother support groups and adherence supporters) facilitated fertility plan discussions. A discrete choice experiment among WLHIV receiving HIV-care at hospitals in Ethiopia and Mozambique also showed peer educators were valued by WLHIV [49]. Training and transferring some fertility plan discussion tasks to mother support groups and adherence supporters can be an effective strategy to address the shortage of trained human resources [31, 50], given that the high client load and insufficient staffing were hindering factors to fertility plan discussions. More importantly, understanding and improving the quality of peer educators could bridge some of the gaps in fertility discussions. Provided that mother support groups and adherence supporters work with a non-governmental organization [46], further integrations into public health facilities might benefit HIV care in our settings.

The lack of recognizing women’s fertility needs by HCPs as well as the lack of initiating conversation by either women or HCPs were barriers to client-centered fertility discussions. This fear may be due to a lack of knowledge regarding effective safer conception strategies and/or stigma towards the fertility of WLHIV. Studies from South Africa and Uganda also documented that reproductive counselling environments were found to be unsupportive of open discussions [26, 51]. There were also tensions between rights-based care for WLHIV and provider’s attitude [26] because HCPs expressed concern about the justification of intentional conception given the risks associated with having children [52]. This tension and perceived stigma in healthcare settings [53] might be the reasons for nondisclosure to their providers of fertility desire or pregnancy during its early stages in our study. Furthermore, WLHIV’s fear of adverse reactions [19, 35] and providers having negative attitudes towards pregnancy of WLHIV [25] and being reluctant to engage women regarding fertility discussions [23] require special attention to meet the reproductive needs of WLHIV. The need for discussing reproductive plans is therefore high because if providers are aware, they can avoid potentially teratogenic drugs [54]. It is important to bridge these gaps because distrust in HCPs might create a negative impact on general HIV care and support [55]. Altogether, this increases the demand for client-centered fertility plan discussion counselling.

Despite men’s positive roles in reproductive counselling [56], inadequate male involvement was a hindering factor for the quality of fertility discussions. Men who consider fertility as a woman’s domain and are unwilling to attend fertility discussions require awareness raising. Resolving mismatched fertility desires among couples is also important. In our study, some women feared disclosing their HIV-positive status to their partners due to a fear of disapproval. Having less control over household and reproductive decision-making among women in Ethiopia might be the reason for the fear of disapproval [57, 58]. Importantly, home-based counselling and testing could fill these gaps if partners were not tested [59]. According to the HCPs, one further critical issue in our study was that if men were HIV-negative and women were HIV-positive, it was more likely to result in divorce. However, in the reverse situation (women being HIV-negative), the couple stayed married, which was also associated with the men engaging in unprotected sex as per the HCPs report. Although further investigation is required, this social factor could be one of the reasons for women being more affected by HIV in Ethiopia [7, 9]. Taken together, improving men’s involvement could benefit general HIV-care, particularly safer conception care and contraceptive use.

According to the provider’s accounts, HCPs counsel discordant couples to avoid having a child because they fear seroconversion and want to avoid complaints. The blame towards the HCPs was related to seroconversion happening during attempting conception while following provider’s advice. In the current study, seroconversions were reported. Given a third of sexually active WLHIV were in discordant relationships [29], effective safer conception strategies had the highest importance. Particularly, pre-exposure prophylaxis is important although currently not provided in many settings [60]. Furthermore, other assisted reproductive strategies are important for discordant couples [45] to achieve their fertility goals. Having guidelines and policies regarding safer conception strategies among WLHIV can reduce the fear associated with seroconversion.

The rigour of this study was established using the criteria of Kitto et al. 2008 [61] and by it being conducted according to consolidated criteria for reporting qualitative research [62]. This study ensured: (i) clarification and justification; (ii) procedural rigour; (iii) representativeness; and (iv) interpretative rigour; (v) reflexivity and evaluative rigour; and (vi) transferability (see Supplementary File 2 for details). Despite this, this paper has an important limitation. While this study provided insights into fertility planning discussions among WLHIV, including unmarried (sexually active) WLHIV would have garnered further understanding of the perspectives specific to unmarried WLHIV.

## Conclusions

There is a need to provide client-centered reproductive healthcare through discussing the personalized fertility plans of HIV-positive people. Policies and guidelines supporting fertility discussions should be available. In particular, safer conception strategies are helpful in reducing the risks associated with having children. Training HCPs regarding safer conception (strategies and effectiveness), improving integration of services along with improved human resources and improving partner involvement to improve reproductive counselling are important. HCPs also need to avoid delaying discussions regarding the fertility issues of their clients. Redirecting tasks such as fertility plan counselling to mother support groups and adherence counsellors could be used as a strategy for HIV-positive people to work towards effective contraceptive use and to achieve safer conception. Improving content and quality of client-centered fertility plan discussions can significantly contribute to having healthy babies and a reduction in HIV transmission. Providing relevant information regarding safer conception strategies and effective contraceptive use in general, according to the fertility needs in particular can help clients retain the information they were given.

## Supplementary information

**Additional file 1: Supplementary file 1.** Data processing and analysis.

**Additional file 2: Supplementary file 2.** The rigour of this study.

## Data Availability

The data that support the findings of this study are available from the Research Centre for Generational Health and Ageing, University of Newcastle, Australia but restrictions apply to the availability of these data, which were used under license for the current study, and so are not publicly available. Data are however available from the authors upon reasonable request and with permission of Research Centre for Generational Health and Ageing, University of Newcastle, Australia at rcgha@newcastle.edu.au. This requirement was imposed by Human Research Ethics Committee of The University of Newcastle, Australia, and the Oromia Regional State Health Bureau Research Ethics Committee, Ethiopia which approved the research protocol.

## Abbreviations

AIDS: Acquired Immunodeficiency Virus
ART: Antiretroviral Therapy
DBS: Dry Blood Sample
HCPs: Healthcare Providers
HIV: Human Immunodeficiency virus
HREC: Human Research Ethics Committee
ICAP: The International Center for AIDS Care and Treatment Programs
IUD: Intrauterine Device
LARC: Long acting reversible contraception
MTCT: Mother To Child Transmissions Of HIV
NGO: Non-Governmental Organization
OCP: Oral Contraceptive pills
PLHIV: People living with HIV
PMTCT: Prevention Of Mother To Child Transmissions Of HIV
SSA: Sub-Saharan Africa
WLHIV: Women Living with HIV

## Footnotes

1 Mum-to-mum group, mother support group, mum-to-mum counsellors, or mother support carers were used interchangeably during interviews. The mother support groups are HIV-positive women working for a non-governmental organization (ICAP).

2 Adherence supporters, adherence counsellors, or case managers were also used interchangeably. They are peer counsellors working for a non-governmental organization (ICAP). They do adherence counselling, patient tracking, education and support.
