# Supplementary material for: “They haven’t asked me. I haven’t told them either”: fertility plan discussions between women living with HIV and healthcare providers in western Ethiopia
Source: Reprod Health. 2020 Aug 17;17:124. doi: 10.1186/s12978-020-00971-2 (PMC7433147; doi:10.1186/s12978-020-00971-2)
Supplement: Supplementary file 1 — Additional file 1: Supplementary file 1. Data processing and analysis. [file 12978_2020_971_MOESM1_ESM.docx]

# **Supplementary file 1: Data processing and analysis**

Interviews were transcribed verbatim in the local language and then translated into English by two healthcare professionals who have master’s degrees. The transcripts were then checked for accuracy by the first author (TRF) by listening to the audio. All transcripts were downloaded into the qualitative management program NVIVO (Version 12. QSR International; 2019) for analysis.

Thematic analysis was used as it is not wedded to a particular epistemology. Braun and Clarke’s (2006) thematic analysis principles (1) were applied as a guide to elucidate the themes. This method was used because it provided a guide for examining the perspectives of participants, highlighting similarities and differences and generating unanticipated insights. It was also considered suitable due to the exploratory nature of the inquiry. An inductive approach to analysis was conducted, using fertility plan discussions as a lens. Transcripts were read in their entirety and repeatedly to immerse the first author in the data and to allow for the initial coding to occur. A list of initial codes (words and phrases) representing a broad array of fertility plan discussions between WLHIV and their HCPs was developed. The coder then performed further coding, through which the broad list of initial codes was condensed into categories. Constant comparisons within and across transcripts and codes were conducted to identify themes. These categories were formed by grouping events, processes and occurrences together. We identified themes through constant comparative analysis, that is, the identification of similarities among and differences between emerging themes. In the process of identifying the major themes, a coding tree was used. Memoing was also used in data generation and data analysis. Records were maintained throughout the process of data collection and analysis. In addition, interviewers took field notes to record socio-demographic characteristics, their interactions and their general observations on the topic. The first author conducted analysis through regular discussions with other authors (MLH and DL). Selected quotes that best described the various categories were included to illustrate major findings while avoiding redundancy. During quoting, WLHIV were identified by code (W#1-W#27), age, number of children they had, number of children desired in the future and whether they discussed their *personal fertility plans* with their HCPs (discussed/not discussed). HCPs were identified by code (HCP#1-HCP#4) to protect their privacy. The quotes from HCPs can be found in Table 2 and Table 3.

**References**

1. Braun V, Clarke V. Using thematic analysis in psychology. Qualitative Research in Psychology. 2006;3(2):77-101.
