# Supplementary material for: “They haven’t asked me. I haven’t told them either”: fertility plan discussions between women living with HIV and healthcare providers in western Ethiopia
Source: Reprod Health. 2020 Aug 17;17:124. doi: 10.1186/s12978-020-00971-2 (PMC7433147; doi:10.1186/s12978-020-00971-2)
Supplement: Supplementary file 2 — Additional file 2: Supplementary file 2. The rigour of this study. [file 12978_2020_971_MOESM2_ESM.docx]

# **Supplementary file 2: The rigour of this study**

The rigour of this study was established using the criteria of Kitto et al. 2008 (1) and by it being conducted according to consolidated criteria for reporting qualitative research (2). Including WLHIV and HCPs who can provide detailed information regarding women’s fertility plan discussions, provided the justification for our research. Comprehensive descriptions of the method of data collection and analysis as well as the setting ensured procedural rigour. Maximum variation sampling (1, 3) of WLHIV (using contraception, not using contraception, pregnant, and breastfeeding) and HCPs was used to improve the representativeness of sampling. Our method of interpretation was enhanced by identifying emerging themes inductively. The credibility of the results was assured by the comprehensiveness and the total immersion of the researcher in the situation. We used representative quotes to illustrate each theme (4). Then, conformability was addressed by having discussions with other authors throughout the analysis and verifying findings of the study.

Regarding reflexivity, the researchers tried to avoid biases and maintain neutrality throughout the research process (data collection, analysis and reporting of findings). Interview-based data collections and theoretical sampling have assured the transferability of the results. Triangulation of findings from different participants and purposive sampling also ensured transferability of the results. Participants were encouraged to continue to describe their feelings and experiences until both the researcher and the participant felt understanding was achieved. Although our study provided crucial insights into fertility discussions between WLHIV and their HCPs, it was not without limitations. Including the unmarried (sexually active) WLHIV would have helped to get a further understanding of the perspectives of unmarried WLHIV.

# **References**

1. Kitto SC, Chesters J, Grbich C. Quality in qualitative research. Med J Aust. 2008;188(4):243-6.

2. Tong A, Sainsbury P, Craig J. Consolidated criteria for reporting qualitative research (COREQ): a 32-item checklist for interviews and focus groups. International journal for quality in health care. 2007;19(6):349-57.

3. Tobin GA, Begley CM. Methodological rigour within a qualitative framework. Journal of advanced nursing. 2004;48(4):388-96.

4. Patton MQ. Qualitative research: Wiley Online Library; 2005.
